# Bond strength and internal adaptation of customized glass fiber posts using different bulk-fill flow resins

**DOI:** 10.4317/jced.57683

**Published:** 2022-03-01

**Authors:** Leticia-Lazzari Fantin, Flávio Simões, Cristiane-de Melo Alencar, Keren-Cristina-Fagundes Jordão-Basso, Suellen-Nogueira-Linares Lima, Matheus-Coelho Bandéca, Mateus-Rodrigues Tonetto

**Affiliations:** 1 Postgraduate Program in Integrated Dental Sciences, University of Cuiabá, Brazil; 2 Department of Restorative Dentistry, Araraquara School of Dentistry, Sao Paulo State University – UNESP

## Abstract

**Background:**

This study aimed to evaluate the bond strength and internal adaptation of customized glass fiber posts using Bulk Fill flowable composite resins (BF) and conventional composite resin.

**Material and Methods:**

Fifty bovine teeth were randomly divided (n=10) according to the following groups: G1 (control): glass fiber posts were adapted to the root canal and luted with Rely-X ARC cement (3M® ESPE); G2: fiber posts smaller than the root canal diameter were customized using Filtek™ Z350 XT (3M® ESPE) conventional composite resin, and luted similarly to Group 1. G3: posts were customized with Tetric N-Ceram Bulk Fill composite resin (Ivoclar Vivadent AG), G4: posts were customized with Filtek™ Bulk Fill Flow (3M® ESPE), and G5: posts were customized with SureFil SDRTM flow (DENTSPLY), respectively. The specimens were submitted to push-out testing and internal adaptation evaluation using optical microscopy. Push-out (MPa) and internal adaptation (%) data were subjected to ANOVA and Tukey’s post-hoc tests (*p* = 5%).

**Results:**

No statistically significant differences were found in both evaluations (*p*<0.05).

**Conclusions:**

Customized glass fiber posts using different bulk fill flowable composites did not affect the post bond-strength and internal adaptation, presenting similar results to customized glass fiber posts using conventional composite or posts with no previous customization.

** Key words:**Dental pulp, composite resins, fiber posts, glass fiber post.

## Introduction

Endodontically-treated teeth with extensive coronal tissue loss require a restorative technique that enhances the restoration retention to the dental remnants (1). Glass fiber post is a better option than metallic post (2), due to its higher resistance to corrosion, is more esthetic, easier to remove from teeth, and presents biomechanical properties more similar to dentin, such as elastic modulus and hardness (3,4).

The success of glass fiber post retention inside the root canal depends on the cementation procedure, once it presents many clinical steps, it may increase the failure chances (5,6). Resin cement and cementation technique may affect the quality of post retention, marginal adaptation, and durability of indirect restorations. Self-adhesive cements have been proposed to simplify the technique owing to their clinical features, such as, simple handling, good flow and good bonding to glass fiber posts (7,8).

The proper retention of glass fiber post inside the root canal depends on the bond strength between post-cement/dentin interface, and a satisfactory adaptation inside the root canal (9). Therefore, teeth with anatomical variations or loss of tooth structure may present larger root canals. Large root canals are more difficult to obtain the post retention closely adapted to the dentin walls, due to the greater thickness of the cementation line (10,11).

Anatomical customization of the fiber post has been recommended as alternative technique in order to obtain a better retention, it proposes to molds the root canal using glass fiber post with composite resin; thus, enabling the formation of a thin and uniform layer of resin cement, favoring the post retention (12). However, previous studies have reported that root reinforcement using conventional composite resin showed low bond-strength values of post to root dentin (9,13), it may have occurred due to an inadequate polymerization in the deeper regions of the root canal (14).

Thus, regarding the teeth with large root canals (15), and bulk-fill composites properties (16,17), this study has proposed an experimental technique of customization and cementation of glass fiber posts using bulk-fill flowable composites. Therefore, the aim of the present study was to evaluate the bond-strength and internal adaptation of customized glass fiber posts using different bulk-fill flowable composites, customized glass fiber posts using conventional composite resin, and fiber posts with no customization. The null hypothesis was: H01: No difference was observed in bond strength of customized glass fiber posts using bulk-fill flowable composite, conventional composite, and non-customized composite. H02: No difference was observed in the internal adaptation between customized glass fiber posts using bulk-fill composite, conventional composite, and posts with no previous customization.

## Material and Methods

- Specimens preparation 

Fifty bovine incisors were selected according to the following inclusion criteria: sound teeth, with crown and root structures preserved, straight roots, absence of cracks and fractures, absence of external root resorption. The teeth were cleansed and kept in 0.1% thymol solution at 4°C, until use.

The roots were standardized and transversely sectioned (15mm length) using a diamond disc at low-speed handpiece under water-cooling, which was confirmed using a digital caliper (Mitutoyo Sul Americana Ltda, Santo Amaro, São Paulo, SP, Brazil) from the apex to the tooth crown.

- Endodontic treatment

The cervical third was irrigated with 2 mL of 2.5% sodium hypochlorite solution (Asfer®, São Caetano do Sul, SP, Brazil), and #15K file (Dentsply - Maillefer, Ballaigues, Switzerland) was inserted in the root canal until it was visible at root apex. The working length was established 1.0 mm short of the total tooth length.

Chemical-mechanical preparation was performed with the crown-down technique. Cervical and middle thirds were prepared using #3 cylindrical burs (LA Axxess™, SybronEndo Corporation, West Collins, Orange, CA, United States). The canals instrumentation was performed using ProTaper Universal (Dentsply-Maillefer, Ballaigues, Switzerland) rotary system up to a F4 instrument, under constant irrigation with 2.5% sodium hypochlorite (Asfer, São Caetano do Sul, SP, Brazil), at each instrument change. The root canals were dried with paper points (Dentsply - Maillefer, Ballaigues, Switzerland), and irrigated with 17% EDTA (Biodinâmica, Paraná, PR, Brazil), for 3 min. After that, the root canals were irrigated with 3 mL of physiological saline solution and dried with absorbent paper points (Dentsply - Maillefer, Ballaigues, Switzerland).

Endodontic obturation was carried out by lateral condensation technique using gutta-percha cone (Dentsply Maillefer, Ballaigues, Switzerland), secondary cones F (Dentsply - Maillefer, Tulsa, Ok, United States), and epoxy-based sealer (AH Plus, Dentsply DeTrey GmbH, Konstanz, Germany). Afterwards, #60 Macspaden compactors (Dentsply - Maillefer, Tulsa, Ok, United States) were used for gutta-percha thermoplastic condensation. Radiographs were taken in the orto-radial direction. Then, the root canal opening was restored with glass ionomer cement (Vidrion R; SS White, Rio de Janeiro, RJ, Brazil), and the specimens were stored at 37°C, 100% relative humidity, for 7 days.

- Post space preparation 

The post space was performed using a #3 Peeso CA drill (Dentsply, Maillefer, Ballaigues, Switzerland), and #3 drill (White Post DC, FGM, Joinville, SC, Brazil), leaving 4 mm of obturation material within the root canals, which was confirmed using radiograph. The canals were irrigated with 2.5% NaOCl at each instrument change and dried with absorbent paper points.

- Glass fiber post preparation 

Pre-fabricated glass fiber posts were cleansed with 70% alcohol for 15s, dried for 15s, adhesive system (Single Bond® Universal - 3M® ESPE, Sumaré, SP, Brazil) was applied according to the manufacture´s recommendations using a microbrush (Microbrush, KG, Sorensen, Barueri, SP, Brazil) and light-cured for 20s (Radi SDI, 1200mW /cm², São Paulo, SP, Brazil).

- Study design 

The teeth were randomly divided (n = 10) according to the following groups:

G1 (control): glass fiber posts that were adapted to the root canal wall were selected. Adhesive system (Single Bond Universal, 3M ESPE, Sumaré, SP, Brazil) was applied and light-cured for 20s into the root canal. The posts were luted with RelyX™ ARC resin cement (3M ESPE, Sumaré, SP, Brazil), handled for 10s according to the manufacture´s recommendations, and inserted into the root canal using a millimeter periodontal probe (Hu-Friedy, Rio de Janeiro, RJ, Brazil). Then, the post was placed inside the root canal, excess was removed by probing, and light-cured for 40s.

G2 (Z350+ARC): fiber posts (0.5 mm) smaller than the root canal diameter were selected. Anatomical customization of the post technique was performed, tooth structure was insulated using glycerin-based gel, then, root canal was molded using the post associated with conventional composite resin (Filtek Z350 XT, 3M ESPE, Sumaré, SP, Brazil). The whole set was light-cured for 40s and removed from the root canal. After that, the post was light-cured outside the root canal for 40s. Glycerine-based gel was removed and customized cementation (3M ESPE, Sumaré, SP, Brazil) was carried out similar to G1.

**Figure 1:**
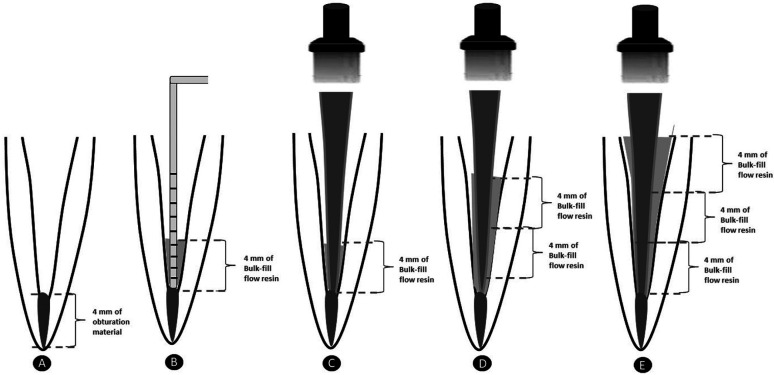
(A) - 4mm of obturator material was left in the apical region of the root canal; (B) - After applying the Bulk-fill flow resin, a millimeter probe was used to check the 4 mm height increment; (C) - The fiberglass post was inserted and cured for 20 sec; (D) - The second 4mm increment was applied and light cured for 20 sec; (E) - The third 4mm increment was applied and cured for 20 sec.

G3 (TN-Ceram BF): fiber posts smaller than the root canal diameter were selected similar to G2. Anatomical customization of the post was carried out, however, using BF composite. Adhesive system was applied and light-cured for 20s into the root canal. The root canal was filled with Tetric N-Ceram Bulk Fill (Ivoclar-vivadent, Barueri, SP, Brazil) flowable composite resin, with 3 increments of 4mm thickness, and light-cured for 20s after each increment, according to the manufactures´ recommendations. After the first increment, the post was inserted and photoactivated for 20 s. Then the other 2 increments were made in the same way (Fig. 1).

G4 (Filtek BF): Similar to G2 and G3, however, customized posts were obtained using Filtek bulk-fill flowable resin (3M® ESPE, Sumaré, SP, Brazil) similar to G3.

G5 (SDRTM BF): Similar to G2, G3 and G4, but, customized posts were obtained using SureFil® SDRTM flowable resin (Dentsply®, York, Pensilvânia, EUA) similar to G3.

- Internal adaptation analysis 

Then, roots were placed inside a PVC matrix and embedded with acrylic resin (Duralay, Illinois, USA). Each root with fiber post was sectioned perpendicularly to the long axis into six 1-mm thick slices. Two slices of cervical, two middle, and two apical thirds were obtained using in a hard tissues cutting machine (Isomet, Buehler Ltd, Lake Bluff, IL, USA) under running water-cooling.15 The coronal side of each slice was identified, and its thickness measured using digital caliper (Mitutoyo Digimatic Caliper, Tokyo, Japan). In order to analyze the post and materials adaptation inside the canal walls, all slices were polished using SofLex PopOn (3M® ESPE, Sumaré, SP, Brazil) abrasive discs system. Then, adhesive interface was analyzed using Optical Microscope (Binocular-Opton), up to 200X magnification.

For quantitative analysis, the interface was assessed with 200X magnification. Each interface slice was divided into 8 areas. A blinded evaluation was performed, the marginal integrity was analyzed using Adobe Photoshop CC 2014 software (Adobe Systems, Mountain View California, CA, USA), and expressed as a percentage of the total length of the internal adaptation.

- Push out testing (bond strength) 

Each slice was submitted to a push-out testing using an electromechanical test machine (Instron 5965 Crp, Canton, United States), at 0.5mm/min speed, until the complete displacement of the post inside the root canal walls using cylinders with different diameters (1.3 mm, 0.9 mm and 0.5 mm). The force required for the post displacement was obtained in N (Newton) and transformed into bond strength (MPa).

- Statistical analysis 

Bond strength (MPa) and internal adaptation (%) data were submitted to ANOVA and Tukey post-hoc tests using a Statistica software (StatSoft, Tulsa, OK, USA) (*p* <0.05).

## Results

- Internal adaptation analysis 

Customized glass fiber post using conventional composite resin presented lower internal adaptation (85.62%) than the other groups, however, no statistically significant differences were found among the groups. Table 1 shows the percentage of internal adaptation of each group.

**Table 1:**
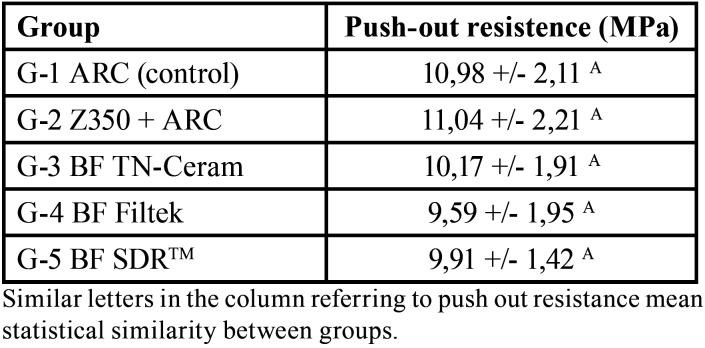
Mean values ± standard deviation of bond strength (MPa) for the groups.

- Push-out bond testing (bond strength) 

The highest bond strength values were found in G1 and G2; however, the analysis of variance showed no statistically significant difference was observed among the groups (*p* <0.05). The mean values and standard deviation of each group are shown in Table 2.

**Table 2:**
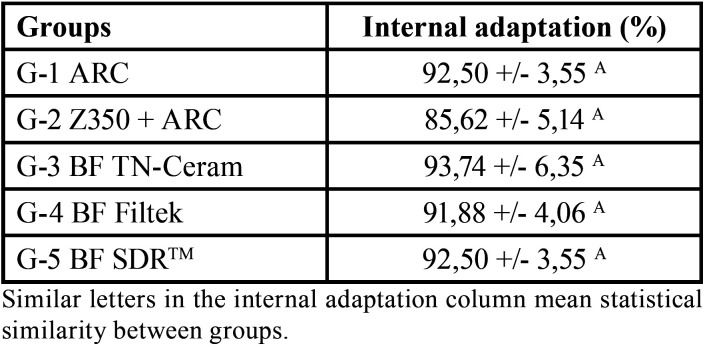
Mean and standard deviation of the internal adaptation of the pins by the percentage (%) of margin adapted in the different groups.

## Discussion

The ideal thickness of resin cement for glass fiber post cementation is still uncertain. However, some previous studies have shown that cements with lower thickness presents better adhesion, thus, higher bond strength of post/cement to root dentin, and less gap formation (9,13). According to the present study, no difference was found in bond strength of cement/ post and internal adaptation among control group, customized groups using bulk-fill or conventional composites. Therefore, both H01 and H02 were accepted and customization using bulk-fill flowable composite may be a promising alternative treatment for endodontically- treated teeth with extensive coronal tissue loss.

Bulk fill composites have been widely used in restorative dentistry, since these composites simplify the clinical procedure, reduce the number of clinical steps, and failure chances (18). Moreover, Bulk fill composites present different properties in comparison to conventional composites, such as, lower tension of polymerization and contraction, which allows increments (up to 4mm) greater than conventional composite increments, thus, reducing the clinical time in posterior restorations (17-20). BF composites present lower shrinkage forces than conventional flowable and high-viscosity composites, thus, BF composites have been indicated to restore high C-factor posterior cavities (21). Furthermore, there are different types and application techniques of BF composites available, with different viscosity, translucency, increment size, performing the dentin filling or even the whole restoration (22). Bulk-fill composites present higher light transmission, regardless of the different filler content and material features (23).

Bakaus *et al*. (2018) (13), evaluated the bond strength of glass fiber posts in large root canals reinforced with BF composites. However, it has used a single increment inside the root canal during customization, and did not use increments up to 4 mm, according to the manufacturer´s recommendations. On the other hand, regarding to reduce a possible effect of the material degree of conversion, the present study has performed the insertion using 3 increments of 4 mm in the customized group using BF. However, no studies have analyzed the incremental technique during posts customization.

Teeth with large root canals require additional techniques to improve the post adaptation inside the root canal (11-13). Clavijo *et al*. has showed that direct and indirect anatomic posts in large root canals can be a good alternative treatment to cast metallic core. Moreover, it was observed that accessory posts presented lower values of fracture resistance (12). According to D’Arcangelo *et al*., the cement thickness negatively affect the post retention when the root canal is larger than the post diameter (11). In the conventional anatomical post technique, post adaptation is performed using composite resin, after canal lubrication, then it is light-cured and coronal restoration is directly carried out in a single session (24). This technique reduces the cementation line, and consequently increase the post intra-canal retention 11, however, many clinical steps are required.

The present study has observed that the bond strength of posts showed no statistically significant difference among the groups. Experimental groups using BF were similar to the control group, and similar to conventional technique of post customization post. It may have occurred due to the standardization of the adhesive system in this study. Single Bond Universal adhesive was used as self-etching strategy in both root dentin and posts treatment. This adhesive system presents silane; thus, it eliminates the post silanization. It simplifies the technique and minimizes clinical time, in accordance to Zaghloul, Elkassas and Haridy (2014) findings (25). In addition, some studies have not observed significant effects of fiber post silanization on the bond strength to dentin (26,27).

Despite of the present study has not observed statistically significant difference in the post internal adaptation among the groups, Jung and Park have showed that bulk-fill flowable composite resins present better marginal adaptation than conventional composites. BF composites present lower polymerization shrinkage and tension, thus, it may have contributed to induce less forces to the root canal margins (28). Conventional composite resin also presented satisfactory marginal adaptation; it may have occurred due to the light conduction potential of the glass fiber post. However, studies have demonstrated a significant reduction of light transmitted according to depth increase in deep cavities (29). On the other hand, the present study has shown that three bulk fill flowable composite resins presented similar results for both bond strength and internal adaptation evaluations. Previous studies have shown positive results of these BF composites in various clinical procedures (19,30).

The limitation of the present study was that no comparison was carried out regarding the different root thirds (cervical, middle and apical) to evaluate the bond strength and internal adaptation of glass fiber post. However, it is relevant to observe that all showed no differences in the comparison of means. Furthermore, studies have reported that endodontic treatment, cementation technique, and the post pre-treatment are factors that may affect the glass fiber posts retention inside root canals (7).

Therefore, BF composites are alternative treatment to optimize clinical time, since these composites present similar bond strength of posts/cement to root dentin, and post internal adaptation similar to conventional composites. Further studies are required to evaluate BF composites adhesion in different root thirds, and to analyze the incremental technique during posts customization.

The present study concluded that customized glass fiber posts using different bulk fill flowable composites did not affect the post bond-strength and internal adaptation, presenting similar results to customized glass fiber posts using conventional composite or posts with no previous customization.
